# Application of a correlation between the lumbar Torg ratio and the area of the spinal canal to predict lumbar stenosis: a study of 420 postmortem subjects

**DOI:** 10.1007/s10195-013-0237-z

**Published:** 2013-04-11

**Authors:** Navkirat S. Bajwa, Jason O. Toy, Nicholas U. Ahn

**Affiliations:** 1 Case Western Reserve University, University Hospitals, 13612 Silver Road, Garfield Heights, OH 44125 USA; 2 Department of Orthopaedics and Rehabilitation, Yale School of Medicine, 800 Howard Ave 1st Floor, New Haven, CT 06519 USA; 3 Department of Orthopaedics, Case Western Reserve University, University Hospitals, 11100 Euclid Ave., Cleveland, OH 44106 USA

**Keywords:** Lumbar Torg ratio, Lumbar stenosis, Morphoanatomy, Canal area

## Abstract

**Background:**

A cervical Torg ratio of 0.8 has been used as a screening tool to determine the presence of cervical spinal stenosis. However, there have been no studies done to define the Torg ratio in the lumbar spine for predicting lumbar spinal stenosis (LSS). Torg ratios have never been correlated with the actual calculated canal area as derived from anatomic specimens. The aim of this study was to provide an analysis of the utility of the lumbar Torg ratio for predicting LSS based on objective measurements of skeletal specimens.

**Materials and methods:**

420 adult skeletal specimens from the Hamann Todd Collection in the Cleveland Museum of Natural History were selected. Digital calipers were used to measure the sagittal diameter (SCD), interpedicular distance, pedicle length, and vertebral body diameter. The canal area at each level was calculated using a geometric formula. A standard distribution curve for canal area and Torg ratio was created, and values that were that is less than the mean minus two standard deviations (SD) below the mean were considered stenotic. Regression analysis was performed to determine if the Torg ratio was correlated with canal area, and if a “below normal” Torg ratio was predictive of LSS.

**Results:**

The Torg ratio for 2SD below the mean was defined as 0.43 at L1, 0.43 at L2, 0.41 at L3, 0.38 at L4, 0.37 at L5. Regression analysis revealed a significant association of the Torg ratio with canal area (*p* < 0.01). A Torg ratio that was less than the mean − 2SD predicted canal stenosis at L2, L3, L4, and L5 (*p* < 0.01). Using a Torg ratio of <0.5 predicted stenosis with a sensitivity of 86 % and specificity of 52 % at all lumbar levels.

**Conclusions:**

Based on the results of our study, we have defined the lower limit of the normal Torg ratio at each level. A Torg ratio of <0.5 predicts LSS and could be a useful radiological tool for LSS screening.

## Introduction

In 1954, Verbiest [1] gave the first clinical description of lumbar spinal stenosis (LSS). Based on various population studies, the incidence of LSS is in the range 5–50 per 10,000 individuals [2, 3]. Shrinkage and loss of disc space due to degeneration with advancing age further aggravates the disease process [4–6]. The prevalence of this disease in the US is expected to increase over the next decade to 18 million [3]. Prior anatomic studies [7–9] have demonstrated that vertebral body diameter increases in older specimens, but these studies are limited in that they have involved only a small number of specimens.

No studies have defined LSS based on morphoanatomic measurements in the normal population. The cervical Torg ratio has been used as a screening tool to determine the presence of cervical spinal stenosis. However, there have been no studies that have attempted to define the Torg ratio in the lumbar spine for predicting LSS. Investigative studies use differing eligibility standards, as there are no widely accepted diagnostic or classification criteria for LSS, which further limits the interpretation of reported findings [10].

A review of the literature suggests that various estimates of the sensitivity and specificity of radiographic diagnosis of lumbar stenosis should be considered inaccurate due to the lack of an independent reference standard [11–17]. Exact measurements that define this condition are needed, as are simple parameters that will accurately predict if LSS is present. The aim of the study described in the present paper was to provide an analysis of the utility of the lumbar Torg ratio for predicting LSS based on objective measurements of skeletal specimens.

## Materials and methods

The Hamann–Todd Osteological Collection in Cleveland, Ohio, contains more than 3,300 treated and dried specimens. Four hundred twenty of these specimens were randomly chosen for examination in no particular order. The specimens in the collection represent individuals who died in Cleveland, Ohio, between the years of 1893–1938. The present study included 314 men and 106 women ranging in age from 20 to 96 years of age. One hundred fifty-eight specimens were of African American ancestry, while the remainder were Caucasian.

**Fig. 1:**
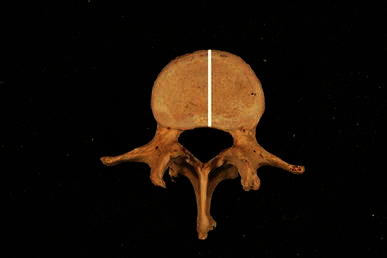
Calculation of the body diameter of the lumbar spine in the anteroposterior plane

**Fig. 2:**
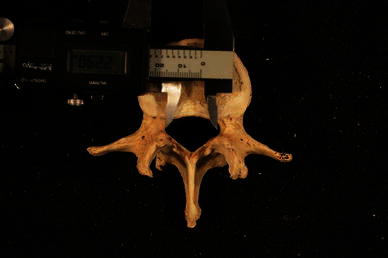
The measurement of IPD after proper alignment of the vertebra

**Fig. 3:**
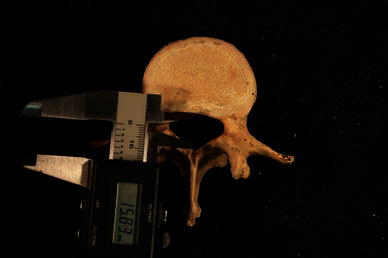
Measuring the SCD from the superior surface of the vertebra

**Fig. 4:**
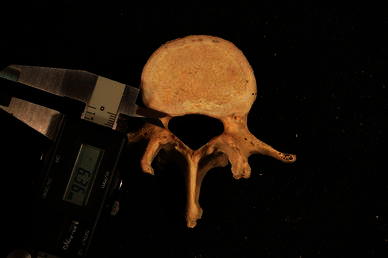
Pedicle length was measured from the superior aspect. The average of both pedicles was used in the study

The gross specimens were then measured subjectively by a single examiner. Digital calipers with a precision of one-hundredth of a millimeter were used for all the measurements. The flat surface of a table edge was used to align each vertebra in the axial plane, and all the measurements were taken from the superior aspect of the vertebrae. The body diameter (VBD) was measured as the anteroposterior distance of each vertebral body (Fig. 1), while the interpedicular distance (IPD) was measured as the minimal distance between the medial surfaces of the pedicles on either side (Fig. 2). The sagittal diameter (SCD) was measured as the maximum anteroposterior distance of the spinal canal of each vertebra (Fig. 3). Pedicle length (PL) was measured starting from the origin of the pedicle from the body to the superior articular facet on either side (Fig. 4). The average was used as the PL.

**Fig. 5:**
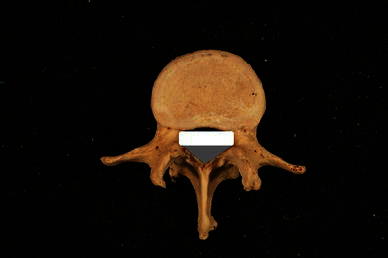
Calculation of the canal area. The total area was calculated as the sum of the area of the *rectangle* (*shaded white*) and the *isosceles triangle* (*shaded gray*)

After the measurements had been taken, the area at each level was calculated using a standardized geometric formula (Fig. 5). To verify these calculations, computerized measurements were done using ImageJ on a random sample of 20 lumbar vertebrae. Results were compared and the kappa value was found. A standard distribution curve for the area at each level was created, and values that were less than the mean minus two standard deviations (SD) were considered stenotic. Stenosis was defined and, for each specimen, the age, sex, and race were also recorded.

The Torg ratio was calculated by dividing the SCD by the VBD. Likewise, Torg ratios that were less than the mean − 2SD were considered to be “below normal.” With this defined, an analysis of deviance using stepwise multivariate linear regression models was performed to determine if the Torg ratio was associated with canal area and if a “below normal” Torg ratio was predictive of lumbar stenosis for each subject. The standard *p*-value cutoff (*p* < 0.05) was used in the study.

## Results

A total of 420 specimens were examined. The full distribution of the specimens by decade of life, sex, and race is shown in Table 1. The percentages of the stenotic specimens in each age group, that are of each sex, and that are of each race are listed in Table 2.

**Table 1:** Age, sex, and racial breakdown of the sampled specimens

Age in years	Number of specimens	Females	Males	White	Black
20–24	15	05	10	02	13
25–34	41	15	26	17	24
35–44	106	25	81	53	53
45–54	105	35	70	68	37
55–64	87	12	75	65	22
65–74	38	09	29	34	04
75–84	22	02	20	19	03
>85	06	03	03	04	02
Total	420	106	314	262	158

**Table 2:** The number of stenotic specimens per age group, sex, and race

Age in years	Number of specimens	Females	Males	White	Black
20–24	1	1	0	0	1
25–34	1	1	0	0	1
35–44	4	2	2	1	3
45–54	5	0	5	4	1
55–64	6	1	5	4	2
65–74	3	2	1	1	2
75–84	2	0	2	2	0
>85	1	0	1	1	0
Total	23	7	16	13	10

LSS was defined at each level as: L1/2 = 2.07 cm^2^; L2/3 = 2.04 cm^2^; L3/4 = 2.00 cm^2^; L4/5 = 1.95 cm^2^; L5/S1 = 1.85 cm^2^. While the SCD dimensions showed very little variation (ranging from 17.3 to 17.7 mm) as we moved from the upper to the lower lumbar levels, the body diameter increased from 31.3 mm at L1 to 34.9 mm at L5. As a result there was a progressive decrease in the Torg ratio from L1 to L5. The mean Torg ratios with their SDs are tabulated in Table 3. The Torg ratio for 2 SD below the mean was defined as 0.43 at L1, 0.43 at L2, 0.41 at L3, 0.38 at L4, 0.37 at L5.

**Table 3:** The mean and the SD value of the lumbar Torg ratio at each level

Lumbar level	Mean Torg ratio	SD	Minimum measurement	Maximum measurement
L1	0.57	0.07	0.40	0.81
L2	0.55	0.06	0.39	0.84
L3	0.53	0.06	0.35	0.77
L4	0.52	0.07	0.33	0.81
L5	0.52	0.08	0.31	0.85

**Fig. 6:**
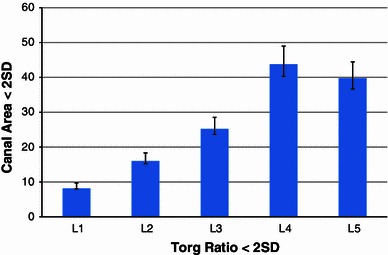
The percentage of specimens with Torg ratios less than the mean − 2SD at each lumbar level (*X* axis) that have lumbar canal stenosis i.e. canal area less than the mean − 2SD (*Y* axis) along with their respective 5 % error bars. A Torg ratio that is 2 SD below the mean predicts canal stenosis at L2, L3, L4, and L5

At all the lumbar levels (L1–L5), a linear stepwise regression analysis revealed a significant association of the Torg ratio with canal area (*p* < 0.01). A Torg ratio that is less than the mean − 2SD predicted canal stenosis at L2, L3, L4, and L5 (*p* < 0.01) with a positive correlation (Fig. 6). The beta and *p* values for each variable are provided in Table 4.

**Table 4:** Beta values and *p* values for age, sex, and race in a linear regression model

Lumbar level	Age		Sex		Race	
	Beta value	*p* value	Beta value	*p* value	Beta value	*p* value
L1	−0.01	0.6	−0.07	0.1	−0.01	0.2
L2	−0.01	0.7	−0.02	0.3	−0.01	0.3
L3	−0.01	0.6	−0.03	0.1	−0.01	0.9
L4	+0.01	0.1	−0.04	0.1	−0.03	0.1
L5	+0.01	0.01	−0.02	0.2	−0.01	0.9

A Torg ratio that is less than the mean − 2SD predicted LSS with a sensitivity of 40 % and specificity of 96 % (Table 5). Using a Torg ratio of <0.55 for the upper lumbar levels (L1 and L2) and <0.5 for lower lumbar levels (L3–L5) predicted stenosis with a sensitivity of 86 % and specificity of 52 % at all lumbar levels (Table 6).

**Table 5:** Torg ratios for 2SD below the mean and their respective sensitivities and specificities in predicting lumbar stenosis

Lumbar level	Lower limit for predicting CLS	Sensitivity %	Specificity %
L1	0.43	12	97
L2	0.43	30	96
L3	0.41	33	97
L4	0.38	50	98
L5	0.37	50	98

**Table 6:** Torg ratios of <0.55 and their respective sensitivities and specificities in predicting lumbar stenosis

Lumbar level	Lower limit for predicting CLS	Sensitivity %	Specificity %
L1	0.55	89	58
L2	0.55	80	40
L3	0.50	80	56
L4	0.50	90	50
L5	0.50	90	53

## Discussion

Lumbar spinal stenosis is defined as a clinical symptom complex that includes low back pain, bilateral lower extremity pain, paresthesias, and other neurologic deficits. It occurs due to anatomic narrowing of the neural pathway through the spine, which may be centrally located in the spinal canal or positioned more laterally in the lateral recesses or neuroforamina. It is postulated that degenerative lumbar stenosis occurs in a high-risk spine with some underlying congenital predisposition [18], but this has not been proven. The anatomic changes result from a cascade of events that include intervertebral disk degeneration, facet joint arthrosis, and hypertrophy of the ligamentum flavum [1, 3]. As a result, the biomechanical characteristics of the spinal segment are altered, which further perpetuates a cycle of degenerative changes.

The lumbar spine has been of great interest to researchers since the early twentieth century [19–22]. A number of studies have tried to define LSS in adults as well as the pediatric population, but a confirmatory diagnosis of LSS is still not possible after more than 50 years of research. Early studies published on the morphometry of the lumbar canal suggested that the spinal index could be used to predict lumbar stenosis [23]. This was proved to be inaccurate by later studies [18, 24, 25].

Torg established the Torg ratio [26] for the cervical spine in order to predict cervical spinal stenosis (CSS) on a lateral radiograph. Since it was first proposed, the Torg ratio has been used as a diagnostic tool for predicting CSS. However, there has been no report in the literature of an attempt to establish a lumbar Torg ratio for predicting LSS. Studies have suggested that the SCD of the lumbar spine is a more accurate measure for predicting LSS than any other measurable parameter [18, 27, 28]. Karantanas et al. [24] conducted a study to investigate correlations of the vertebral dimensions with somatometric parameters in 100 patients presenting with low back pain. They concluded that the AP diameter was the only measurement that could be used to estimate LSS, and was independent of other somatometric parameters. It has also been suggested that measurements in the transverse plane are independent of measurements in the AP plane [29]. As a result, it makes sense to establish a Torg ratio for the lumbar spine that takes into account the AP diameter of the lumbar spinal canal and the AP diameter of the lumbar vertebral body. De Graaf et al. [17] suggested that radiological studies have inherent inaccuracies, and this has been shown to be true by a number of radiological studies with conflicting conclusions [11, 12, 30–32]. Thus, a morphoanatomical study to establish a definite Torg ratio criterion for lumbar stenosis is warranted.

In a study by Eisenstein [18], 45 of the 2,166 lumbar vertebrae of adult skeletons that were measured were found to be stenotic. The mid-sagittal diameter was the significantly reduced dimension and persistently predicted spinal stenosis. They reported that on lateral plain radiography, the overall average lower limit of the mid-sagittal diameter was 15 mm. A spinal index ratio of 1:4.5, the lower limit for a normal (“stenotic”) canal, predicted 11 % of vertebrae as stenotic, an overestimation. The ratio of the AP diameter of the spinal canal to the AP VBD was never calculated. Amonoo-kuofi [33, 34] extensively researched the morphology of lumbar spines in a negroid population in various studies. The mean SCD and AP VBD were defined morphoanatomically in 122 cadaveric spines. He reported that the ratio of the AP diameter of the canal to the AP diameter of the vertebral body was highest at L1 (0.6), and had a constant value of 0.5 from L2 to L5. However, due to the wide variation in the values of these findings, no definite conclusions could be drawn. The canal area was not calculated, and these findings were never standardized and correlated with the canal area to define lumbar stenosis.

Some studies have established other ratios comparing the IPD diameter or the PL with the vertebral body diameter [9, 35]. However, there have been no follow-up studies of these ratios to investigate their efficacy for predicting LSS.

All of the above studies were done on relatively small samples, meaning that it has not been possible to define a set standard or bony lumbar stenosis. The major focus in these studies was on a single aspect of stenosis (i.e., either looking at the cross-sectional area alone or at the IPD and SCD). These anatomic studies were performed on African and European populations. None of these studies managed to establish a lumbar Torg ratio for predicting lumbar stenosis in an average American population. In our study, we morphoanatomically compared a much wider array of representatives of the general American population, ranging from adolescents to very old individuals. The inherent inaccuracies of investigations done via the radiological techniques of MR or CT were nullified in our study.

As this study was a retrospective, cadaveric study, there are some inherent limitations to it. Ideally, we would perform a prospective cohort study following a large group of patients with serial imaging studies and autopsy analysis after death. Such a study would provide the most satisfactory answers to the questions addressed in the present study. The problem is that such a study would be logistically difficult and financially prohibitive. There is always some component of soft tissue involved in the overall pathogenesis [2, 3] which, due to the innate restrictions of this study, cannot be taken into account. We would need a clinical study to assess the correct diagnostic levels. This study is a statistical collection of skeletal data where we arbitrarily used 2SD as the threshold; this would vary according to the signs and symptoms of each patient in a clinical study. A diagnostic threshold can only be defined using either X-ray or CT/MR images for reproducible use in a clinical scenario.

Although, from a biological standpoint, the nutrients received from foods have not changed significantly over the past 100 years, and bone quality and structure have remained essentially the same [36], the last century has seen the emergence of obesity as a frequent cause of several significant health problems. Thus, the findings of this study are limited in the context that BMI cannot be calculated due to a lack of morphometric data, meaning that obesity and its effect on the skeletal system (especially the spine) cannot be studied due to inherent restrictions of the study design.

In conclusion, based on our study of a large population of adult skeletal specimens, we have defined a statistical Torg ratio for predicting LSS at each level. The morphology of the lumbar spine varies considerably from one lumbar level to another as well as with advancing age, but since the Torg ratio is a ratio, it is not influenced by changes in the AP dimensions. As a result, it was possible to define a lower limit for the Torg ratio for all 5 lumbar vertebrae. This study encompassed a much larger population of adult American individuals and a greater range of changes to the lumbar region due to the development of stenosis than any previous study in this field. What this skeletal study has shown is that there is a definite correlation of small canal area with low Torg ratio. Also, the results indicate that lumbar Torg ratios tend to be lower than cervical Torg ratios. However, the results of this study should be confirmed by clinical radiological studies.

This study considered all aspects of lumbar stenosis and correlated canal cross-sectional area with predictive parameters of bony anatomy. These parameters were found to be correlated with the lumbar Torg ratio with varying sensitivities and specificities. This study addressed the pitfalls of previous anatomic studies that placed special emphasis on the radiodiagnosis of LSS.
